# Supplementary material for: The Atmospheric Pressure Capillary Plasma Jet Is Well‐Suited to Supply H2O2 for Plasma‐Driven Biocatalysis
Source: ChemistryOpen. 2025 Jun 22;14(9):e202500057. doi: 10.1002/open.202500057 (PMC12409826; doi:10.1002/open.202500057)
Supplement: Supplementary file 1 — Supplementary Material [file OPEN-14-e202500057-s001.pdf]

Supplemental Material

The Atmospheric Pressure Capillary Plasma Jet Is Well-Suited to Supply H_2O_2 for Plasma-Driven Biocatalysis

Tim Dirks[a], Davina Stoesser[a], Steffen Schüttler[b], Frank Hollmann[c], Judith Golda[b], Julia E. Bandow[a]

[a]Applied Microbiology, Faculty of Biology and Biotechnology, Ruhr University Bochum, Germany

[b]Plasma Interface Physics, Faculty of Physics and Astronomy, Ruhr University Bochum, Germany

[c]Department of Biotechnology, Delft University of Technology, Delft, The Netherlands

Supplementary Table 1: Calculations of r*Aae*UPO concentrations and TON using HA403 M beads and the capillary plasma jet with 6400 ppm H_2O in feed gas.

**Enzyme loading of beads in immobilization**	
amount of beads [mg]	500
total volume [ml]	5
r*Aae*UPO concentration [nM]	400
r*Aae*UPO amount [nmol]	2
maximum loading of beads [nmol/100 mg beads]	0.4
binding efficiency [%]	44.68
actual loading of beads [nmol/100 mg]	0.18
**Final concentrations of r*Aae*UPO in reaction**	
volume used for reactor [ml]	1.5
amount of beads in reactor [mg]	150
amount r*Aae*UPO in reactor [nmol]	0.27
reaction volume [ml]	5
r*Aae*UPO concentration in reaction [nM]	53.62
**TON calculations after 40 min**	
product concentration [mM]	1.73
turnover number	32,265
**TON calculations after 80 min**	
product concentration [mM]	2.37
turnover number	44,200

Supplementary Table 2: Calculations of r*Aae*UPO concentrations and TON using EA403 M beads the capillary plasma jet with 6400 ppm H_2O in feed gas.

**Enzyme loading of beads in immobilization**	
amount of beads [mg]	500
total volume [ml]	5
r*Aae*UPO concentration [nM]	400
r*Aae*UPO amount [nmol]	2
maximum loading of beads [nmol/100 mg beads]	0.4
binding efficiency [%]	59.95
actual loading of beads [nmol/100 mg]	0.24
**Final concentrations of r*Aae*UPO in reaction**	
volume used for reactor [ml]	1.5
amount of beads in reactor [mg]	150
amount r*Aae*UPO in reactor [nmol]	0.36
reaction volume [ml]	5
r*Aae*UPO concentration in reaction [nM]	71.95
**TON calculations after 40 min**	
product concentration [mM]	1.81
turnover number	25,261
**TON calculations after 80 min**	
product concentration [mM]	2.45
turnover number	34,097

Supplementary Table 3: Calculations of r*Aae*UPO concentrations and TON using ECR8309F beads the capillary plasma jet with 6400 ppm H_2O in feed gas.

**Enzyme loading of beads in immobilization**	
amount of beads [mg]	500
total volume [ml]	5
r*Aae*UPO concentration [nM]	400
r*Aae*UPO amount [nmol]	2
maximum loading of beads [nmol/100 mg beads]	0.4
binding efficiency [%]	42.33
actual loading of beads [nmol/100 mg]	0.17
**Final concentrations of r*Aae*UPO in reaction**	
volume used for reactor [ml]	1.5
amount of beads in reactor [mg]	150
amount r*Aae*UPO in reactor [nmol]	0.25
reaction volume [ml]	5
r*Aae*UPO concentration in reaction [nM]	50.80
**TON calculations after 40 min**	
product concentration [mM]	1.66
turnover number	32,708

Supplementary Table 4: Calculations of r*Aae*UPO concentrations and TON using ECR8285 beads the capillary plasma jet with 6400 ppm H_2O in feed gas.

Enzyme loading of beads in immobilization	
amount of beads [mg]	500
total volume [ml]	5
r*Aae*UPO concentration [nM]	400
r*Aae*UPO amount [nmol]	2
maximum loading of beads [nmol/100 mg beads]	0.4
binding efficiency [%]	44.40
actual loading of beads [nmol/100 mg]	0.18
**Final concentrations of r*Aae*UPO in reaction**	
volume used for reactor [ml]	1.5
amount of beads in reactor [mg]	150
amount r*Aae*UPO in reactor [nmol]	0.27
reaction volume [ml]	5
r*Aae*UPO concentration in reaction [nM]	53.28
**TON calculations after 40 min**	
product concentration [mM]	1.08
turnover number	20,338

Supplementary Table 5: Calculations of the linear slope of r*Aae*UPO in plasma-driven biocatalysis using different carriers for immobilization and the capillary plasma jet with 6400 ppm H_2O in feed gas.

Linear slope [µM (*R*)-1-PhOl min^{-1}]	
HA403 M	49.03
EA403 M	54.57
ECR8309F	47.32
ECR8285	31.90

Supplementary Table 6: Calculations of TTN using r*Aae*UPO immobilized on HA403 M beads in long-term biocatalysis with the capillary plasma jet. 6400 ppm H_2O was added to the feed gas and buffer exchange was performed every 10 min.

**Enzyme loading of beads in immobilization**	
amount of beads [mg]	500
total volume [ml]	5
r*Aae*UPO concentration [nM]	400
r*Aae*UPO amount [nmol]	2
maximum loading of beads [nmol/100 mg beads]	0.4
binding efficiency [%]	43.00
actual loading of beads [nmol/100 mg]	0.17
**Final concentrations of r*Aae*UPO in reaction**	
volume used for reactor [ml]	4
amount of beads in reactor [mg]	400
amount r*Aae*UPO in reactor [nmol]	0.69
reaction volume [ml]	5
r*Aae*UPO concentration in reaction [nM]	137.61
**TTN calculation**	
product [µmol]	83.43
total turnover number	122,138

Supplementary Table 7: Calculations of TTN using r*Aae*UPO immobilized on HA403 M beads in long-term biocatalysis with the capillary plasma jet. 6400 ppm H_2O was added to the feed gas and buffer exchange was performed every 5 min.

**Enzyme loading of beads in immobilization**	
amount of beads [mg]	500
total volume [ml]	5
r*Aae*UPO concentration [nM]	400
r*Aae*UPO amount [nmol]	2
maximum loading of beads [nmol/100 mg beads]	0.4
binding efficiency [%]	47.14
actual loading of beads [nmol/100 mg]	0.19
**Final concentrations of r*Aae*UPO in reaction**	
volume used for reactor [ml]	4
amount of beads in reactor [mg]	400
amount r*Aae*UPO in reactor [nmol]	0.75
reaction volume [ml]	5
r*Aae*UPO concentration in reaction [nM]	150.85
**TTN calculations**	
product [µmol]	102.13
total turnover number	138,777

Supplementary Table 8: Calculations of TTN using r*Aae*UPO immobilized on HA403 M beads in long-term biocatalysis with the capillary plasma jet. 1280 ppm H_2O was added to the feed gas and buffer exchange was performed every 5 min.

**Enzyme loading of beads in immobilization**	
amount of beads [mg]	500
total volume [ml]	5
r*Aae*UPO concentration [nM]	400
r*Aae*UPO amount [nmol]	2
maximum loading of beads [nmol/100 mg beads]	0.4
binding efficiency [%]	43.85
actual loading of beads [nmol/100 mg]	0.17
**Final concentrations of r*Aae*UPO in reaction**	
volume used for reactor [ml]	4
amount of beads in reactor [mg]	400
amount r*Aae*UPO in reactor [nmol]	0.70
reaction volume [ml]	5
r*Aae*UPO concentration in reaction [nM]	140.32
**TTN calculations**	
product [µmol]	122.06
total turnover number	174,209

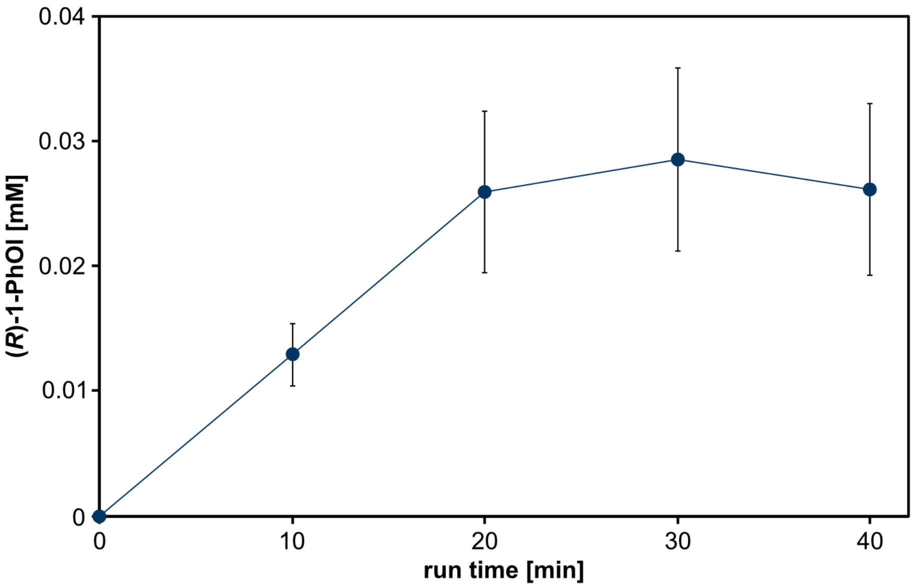

Supplementary Figure 1: Plasma-driven biocatalysis with the capillary plasma jet using rAaeUPO immobilized on ReliZyme EP403 M beads. Conversion of the substrate ETBE utilized H_2O_2 from direct plasma treatment of the r*Aae*UPO immobilized on ReliZyme EP403 M. Reaction solution contained 5 ml potassium phosphate buffer (100 mM, pH 7) with 50 mM ETBE. Plasma treatment was performed using the capillary plasma jet as described above, with a water concentration of 6400 ppm in the feed gas at 6 W plasma power. Every 10 min, aliquots were withdrawn for product analysis by GC. Means and standard deviations reflect three experiments.

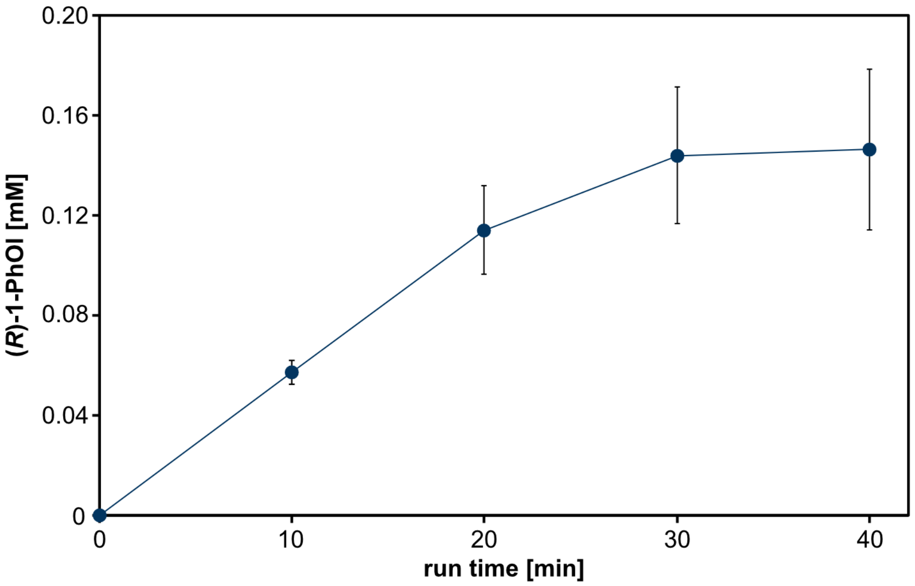

Supplementary Figure 2: Plasma-driven biocatalysis with the capillary plasma jet using rAaeUPO immobilized on ReliZyme HFA403 M beads. Conversion of the substrate ETBE utilized H_2O_2 from direct plasma treatment of the r*Aae*UPO immobilized on ReliZyme HFA403 M. Reaction solution contained 5 ml potassium phosphate buffer (100 mM, pH 7) with 50 mM ETBE. Plasma treatment was performed using the capillary plasma jet as described above, with a water concentration of 6400 ppm in the feed gas at 6 W plasma power. Every 10 min, aliquots were withdrawn for product analysis by GC. Means and standard deviations represent three experiments.

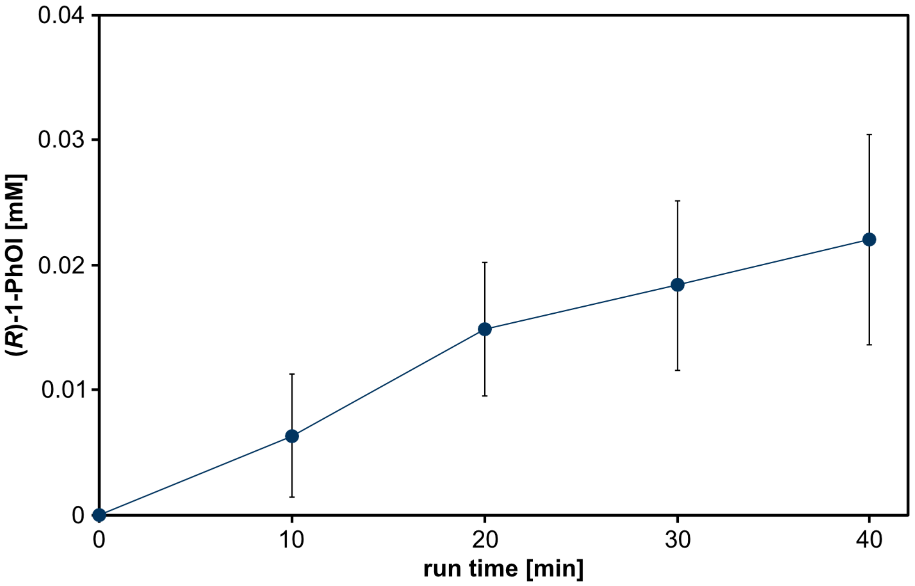

Supplementary Figure 3: Plasma-driven biocatalysis with the capillary plasma jet using rAaeUPO immobilized on ReliZyme BU403 M beads. Conversion of the substrate ETBE utilized H_2O_2 from direct plasma treatment of the r*Aae*UPO immobilized on ReliZyme BU403 M. Reaction solution contained 5 ml potassium phosphate buffer (100 mM, pH 7) with 50 mM ETBE. Plasma treatment was performed using the capillary plasma jet as described above, with a water concentration of 6400 ppm in the feed gas at 6 W plasma power. Every 10 min, aliquots were withdrawn for product analysis by GC. Means and standard deviations reflect three experiments.

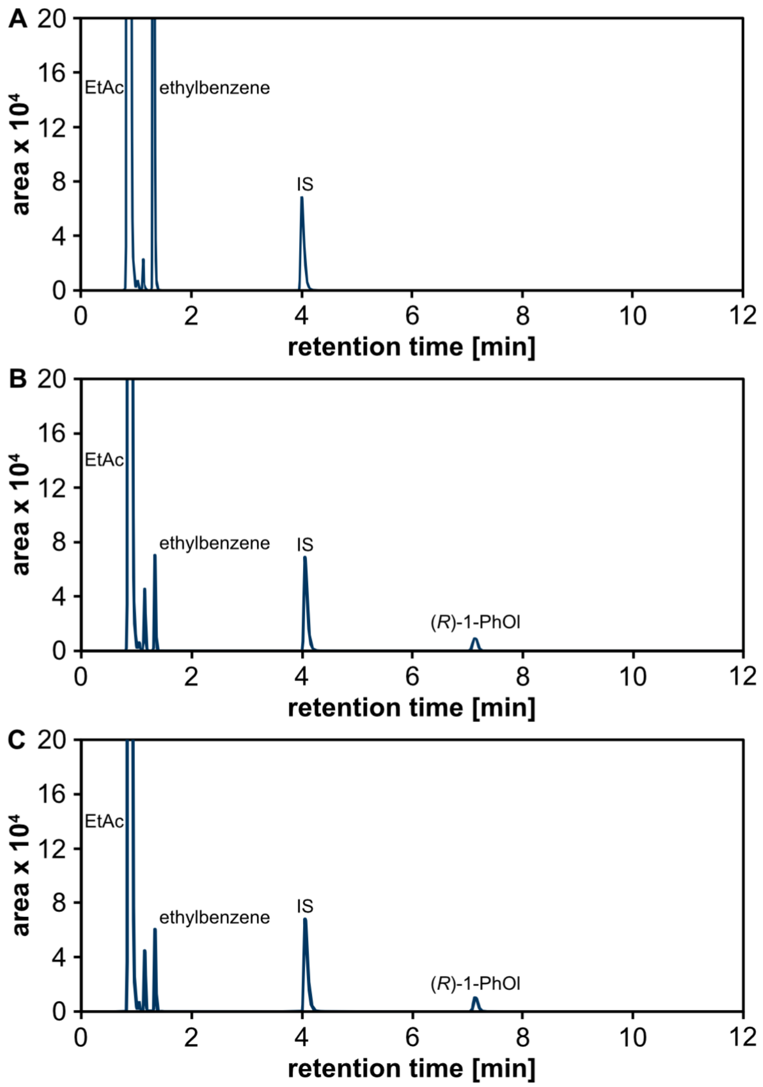

Supplementary Figure 4: Substrate and product stability during plasma-driven biocatalysis. r*Aae*UPO on ReliZyme beads was operated in 5 ml potassium phosphate buffer (100 mM, pH 7) with 50 mM ethylbenzene serving as substrate. The capillary plasma jet was operated with 6400 ppm water in the feed gas (6 W plasma power). To assess substrate and product stability, after 20 min of continuous plasma-driven biocatalysis the reaction solution was extracted with ethylacetate (EtAc). 1-Octanol was added as internal standard (IS). GC-FID analysis was used to assess concentrations of substrate, product ((*R*)-1-PhOl), and potential side or degradation products. A: no plasma was ignited (no H_2O_2 production). B: reaction with r*Aae*UPO immobilized on HA403 M beads. C: reaction with r*Aae*UPO immobilized on EA403 M beads.

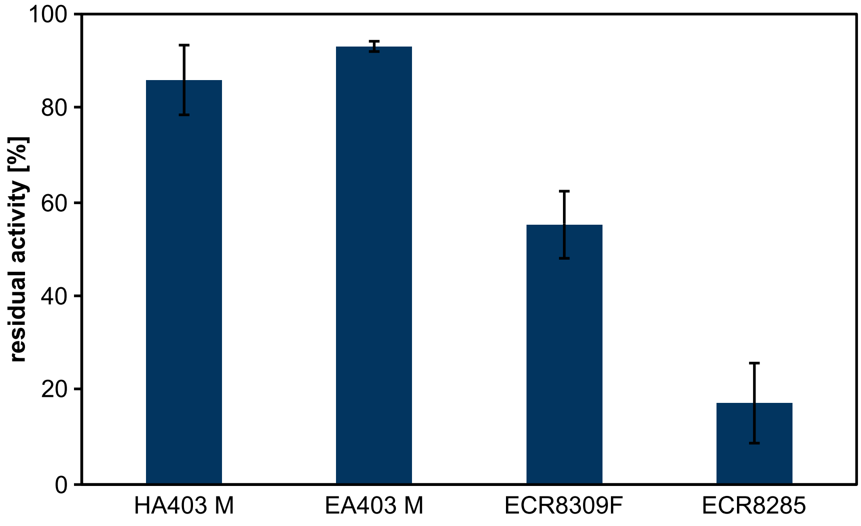

Supplementary Figure 5: Residual activity of r*Aae*UPO on ReliZyme (HA403 M, EA403 M) and Purolite (ECR8309F and ECR8285) beads after 40 min plasma-driven biocatalysis. After biocatalysis, enzyme-loaded beads were recovered and washed thrice with potassium phosphate buffer (100 mM, pH 7). Enzyme activity was determined using 2.5 mM 2,2'-azino-bis(3-ethylbenzothiazoline-6-sulfonic acid) (ABTS), 1 mM H_2O_2 and 50 mM citrate. Samples were shaken during turnover to ensure sufficient substrate supply. Every two minutes in a total of ten minutes reaction time, aliquots of 100 µl were withdrawn and measured at 405 nm using a microplate reader (Biotek Epoch). Enzyme activity was calculated based on the linear slope of the kinetic. Means and standard deviations represent three experiments.

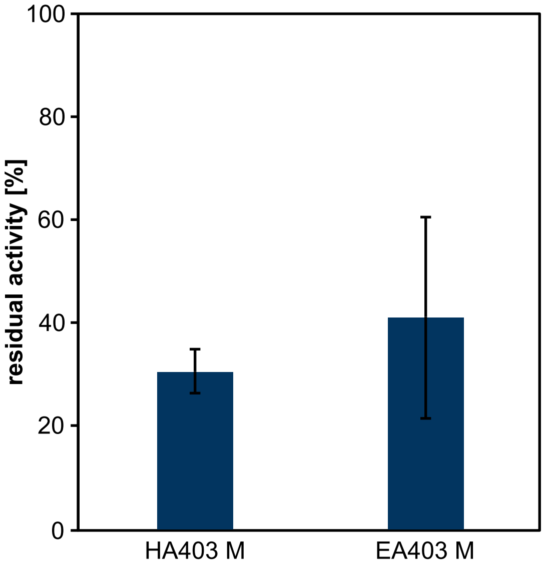

Supplementary Figure 6: Residual activities of r*Aae*UPO on ReliZyme beads after 80 min plasma-driven biocatalysis with the capillary plasma jet. Biocatalysis was performed with the capillary plasma jet and a water concentration of 6400 ppm in the feed gas at 6 W plasma power for a run time of 80 min. After biocatalysis, enzyme-loaded beads were recovered and washed thrice with potassium phosphate buffer (100 mM, pH 7). Enzyme activity was determined using 2.5 mM 2,2'-azino-bis(3-ethylbenzothiazoline-6-sulfonic acid) (ABTS), 1 mM H_2O_2 and 50 mM citrate. Samples were shaken during turnover to ensure sufficient substrate supply. Every two minutes in a total of ten minutes reaction time, aliquots of 100 µl were removed and measured at 405 nm using a microplate reader (Biotek Epoch). Enzyme activity was calculated based on the linear slope of the kinetic. Means and standard deviations reflect three experiments.

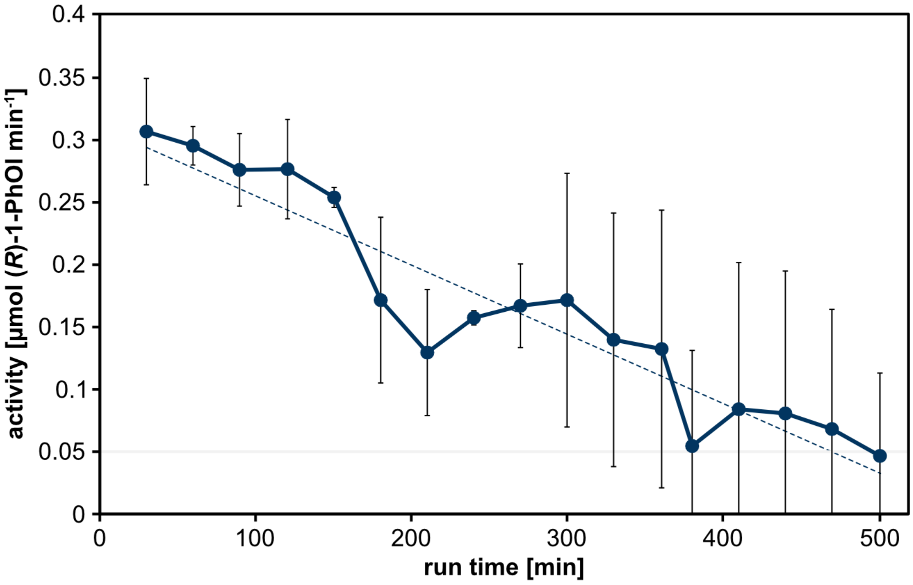

Supplementary Figure 7: Product formation per minute in long-term experiments using 6400 ppm H_2O in the feed gas. Activity of r*Aae*UPO immobilized on HA403 M beads is plotted as a function of plasma-driven biocatalysis run time. Every 10 min, the complete reaction solution was exchanged and product formation was analyzed by GC measurement. Means and standard deviations reflect three experiments.

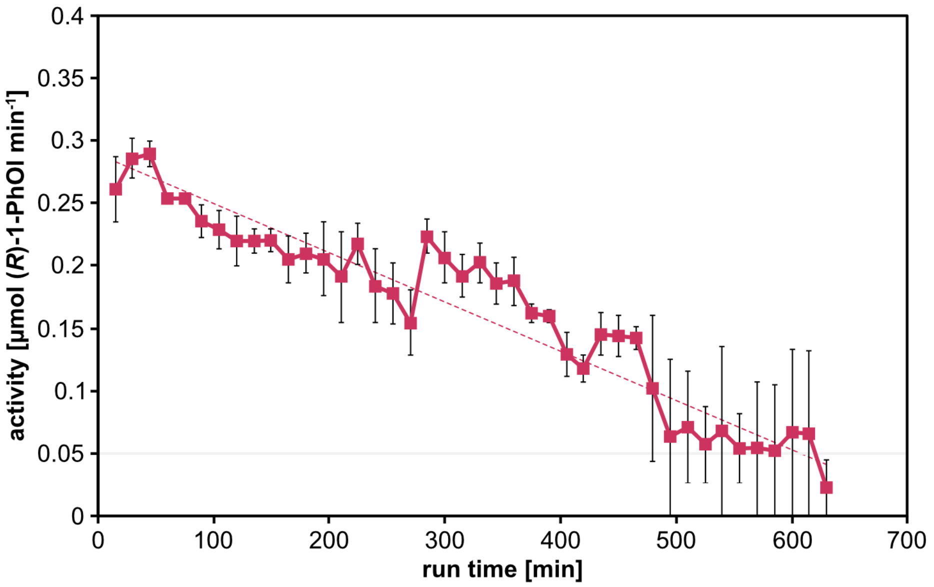

Supplementary Figure 8: Product formation per minute in long-term experiments using 6400 ppm H_2O in the feed gas. Activity of r*Aae*UPO immobilized on HA403 M beads is plotted as a function of plasma-driven biocatalysis run time. Every 5 min, the complete reaction solution was exchanged and product formation was analyzed by GC measurement. Means and standard deviations reflect three experiments.

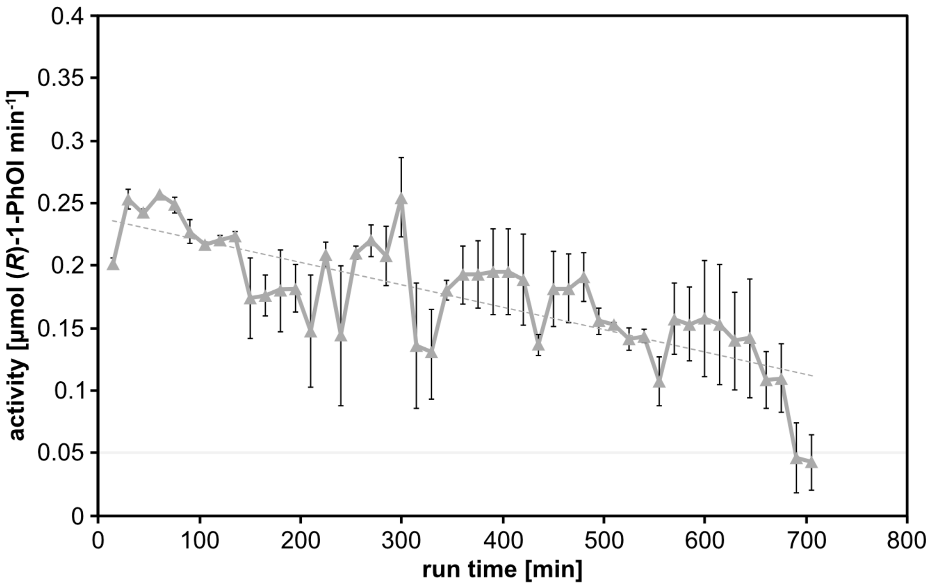

Supplementary Figure 9: Product formation per minute in long-term experiments using 1280 ppm H_2O in the feed gas. Activity of r*Aae*UPO immobilized on HA403 M beads is plotted as a function of plasma-driven biocatalysis run time. Every 5 min, the complete reaction solution was exchanged and product formation was analyzed by GC measurement. Means and standard deviations represent three experiments.
